# Dual-time-point 68Ga-FAPI PET/CT in gastrointestinal malignancies: head-to-head comparison with 18 F-FDG for lesion detection and patient-level assessment

**DOI:** 10.1007/s12149-026-02249-2

**Published:** 2026-06-25

**Authors:** Hasan Pala, Murat Tuncel, Suayib Yalcin, Erdem Karabulut, Nezih Akkapulu, Timucin Erol, Omer Ugur

**Affiliations:** 1 Department of Nuclear Medicine, Dr. Abdurrahman Yurtaslan Ankara Oncology Training and Research Hospital, Ankara, Türkiye; 2 Department of Nuclear Medicine, Hacettepe University Faculty of Medicine, Ankara, Türkiye; 3 Department of Medical Oncology, Hacettepe University Faculty of Medicine, Ankara, Türkiye; 4 Department of Biostatistics, Hacettepe University Faculty of Medicine, Ankara, Türkiye; 5 Department of General Surgery, Hacettepe University Faculty of Medicine, Ankara, Türkiye

**Keywords:** Gastrointestinal Malignancies, 18F-FDG PET/CT, Dual-phase FAPI imaging, Peritoneal Carcinomatosis, Lesion Detection, Patient-Level Assessment

## Abstract

**Objectives:**

To compare the diagnostic performance of 18 F-Fluorodeoxyglucose (FDG) and 68Ga-fibroblast activation protein inhibitor (FAPI) positron emission tomography/computed tomography (PET/CT) in gastrointestinal malignancies, with particular emphasis on lesion detection, peritoneal disease assessment, and the potential added value of dual-time-point FAPI imaging.

**Methods:**

Thirty-eight patients with histologically confirmed gastrointestinal cancers who underwent both FDG and FAPI PET/CT within a 4-week interval were retrospectively analyzed. FAPI PET/CT images were acquired at 10 and 60 min after tracer injection. Patient-level analyses (staging/restaging accuracy, impact on management) and lesion-based analyses (detection rate, characterization, maximum standardized uptake value [SUVmax] dynamics, lesion size) were evaluated against follow-up data as the reference standard.

**Results:**

FAPI PET/CT demonstrated superior agreement with the reference standard compared with FDG PET/CT for staging and restaging (κ = 0.91 vs. 0.32). On lesion-based analysis, FAPI showed markedly higher detection rate for malignant lesions (86% vs. 34%) and fewer indeterminate findings. Median peritoneal carcinomatosis index score and the number of detected malignant lesions per patient were significantly higher with FAPI PET/CT (*p* < 0.001). Dual-time-point FAPI imaging revealed a significant increase in SUVmax from early to later time points in malignant lesions, whereas benign lesions showed no significant change. Larger lesions (> 10 mm) demonstrated more pronounced delayed uptake, and greater early-to-delayed SUVmax increases were independently associated with malignancy, suggesting a potential role for dynamic uptake patterns in lesion characterization.

**Conclusions:**

Dual-time-point 68Ga-FAPI PET/CT demonstrated superior lesion detectability compared with FDG PET/CT in gastrointestinal malignancies and may help reduce diagnostic uncertainty. These findings suggest that dual-time-point FAPI imaging may provide incremental value for lesion characterization and support its complementary role in oncologic imaging.

## Introduction

Gastrointestinal malignancies continue to represent a significant global health issue and remain among the most prominent causes of cancer-related morbidity and mortality worldwide [1, 2]. Accurate staging and restaging are pivotal to guiding treatment decisions and estimating prognosis, particularly in patients with advanced or recurrent disease. From a clinical perspective, the identification of peritoneal metastases is especially critical, as peritoneal involvement is consistently associated with unfavorable outcomes and often leads to a fundamental change in therapeutic approach [3, 4].

18 F-Fluorodeoxyglucose (FDG) positron emission tomography/computed tomography (PET/CT) is frequently used in the management of gastrointestinal cancers to evaluate metastatic spread and suspected recurrence [5]. However, FDG PET/CT may show limited sensitivity in specific tumor subtypes, including mucinous or low-grade malignancies as well as small-volume disease, particularly within the peritoneal cavity [6]. In addition, partial-volume effects and physiological abdominal uptake may further impair lesion detectability, especially for small lesions [7].

Fibroblast activation protein inhibitor (FAPI) PET/CT has recently emerged as a promising molecular imaging technique for targeting cancer-associated fibroblasts in the tumor microenvironment [8, 9]. FAP expression is abundant in the stromal component of many gastrointestinal malignancies and plays a central role in tumor growth, invasion, and extracellular matrix remodeling [10]. Accordingly, FAPI PET/CT has demonstrated encouraging results in detecting both primary and metastatic lesions in various gastrointestinal cancers owing to its low physiological background activity and high tumor-to-background contrast [11–13].

Several head-to-head comparative studies have suggested that FAPI PET/CT may outperform FDG PET/CT, particularly in the detection of recurrent disease and peritoneal metastases [11–14]. Recent meta-analyses support these findings as indicating that FAPI PET/CT has superior sensitivity for small-volume and peritoneal disease [15, 16]. Most studies rely on imaging protocols performed at a single time point, mainly emphasizing patient detection rates. Consequently, this approach provides only limited insight into tracer kinetics or into detailed lesion features, such as morphology, size, or regional distribution.

Given the pharmacokinetic properties of FAPI tracers, the timing of acquisition may affect lesion detectability and the tumor-to-background contrast. Nevertheless, the clinical significance of early versus delayed imaging remains inadequately defined [17]. Furthermore, due to partial volume effects, lesion size is a critical determinant of PET/CT imaging [7]. However, its impact on the comparative diagnostic performance of FDG and FAPI PET/CT in gastrointestinal malignancies remains uninvestigated. Accurate imaging-based assessment of peritoneal tumor burden, including estimation of the peritoneal carcinomatosis index (PCI), is increasingly recognized as clinically relevant for patient selection and surgical planning [18, 19].

Therefore, this retrospective study aimed to compare FDG and FAPI PET/CT in patients with gastrointestinal malignancies undergoing staging or restaging, with respect to lesion detection, identification of recurrent disease, tumor staging, and assessment of peritoneal involvement. In addition, we evaluated the impact of dual-time-point FAPI PET/CT imaging at 10th and 60th minutes post-injection and explored the influence of lesion characteristics on diagnostic performance.

## Materials and methods

### Study design and ethical approval

This single-center retrospective study was approved by the Ethics Committee for Scientific Research in Health Sciences at Hacettepe University Faculty of Medicine (study ID: SBA 24/450) and was conducted in accordance with the Declaration of Helsinki. All participants provided written informed consent before the PET/CT scans, permitting the use of anonymized clinical and imaging data for future research. Data were obtained during routine clinical care, without additional procedures performed for research purposes.

### Patient population

**Fig. 1:**
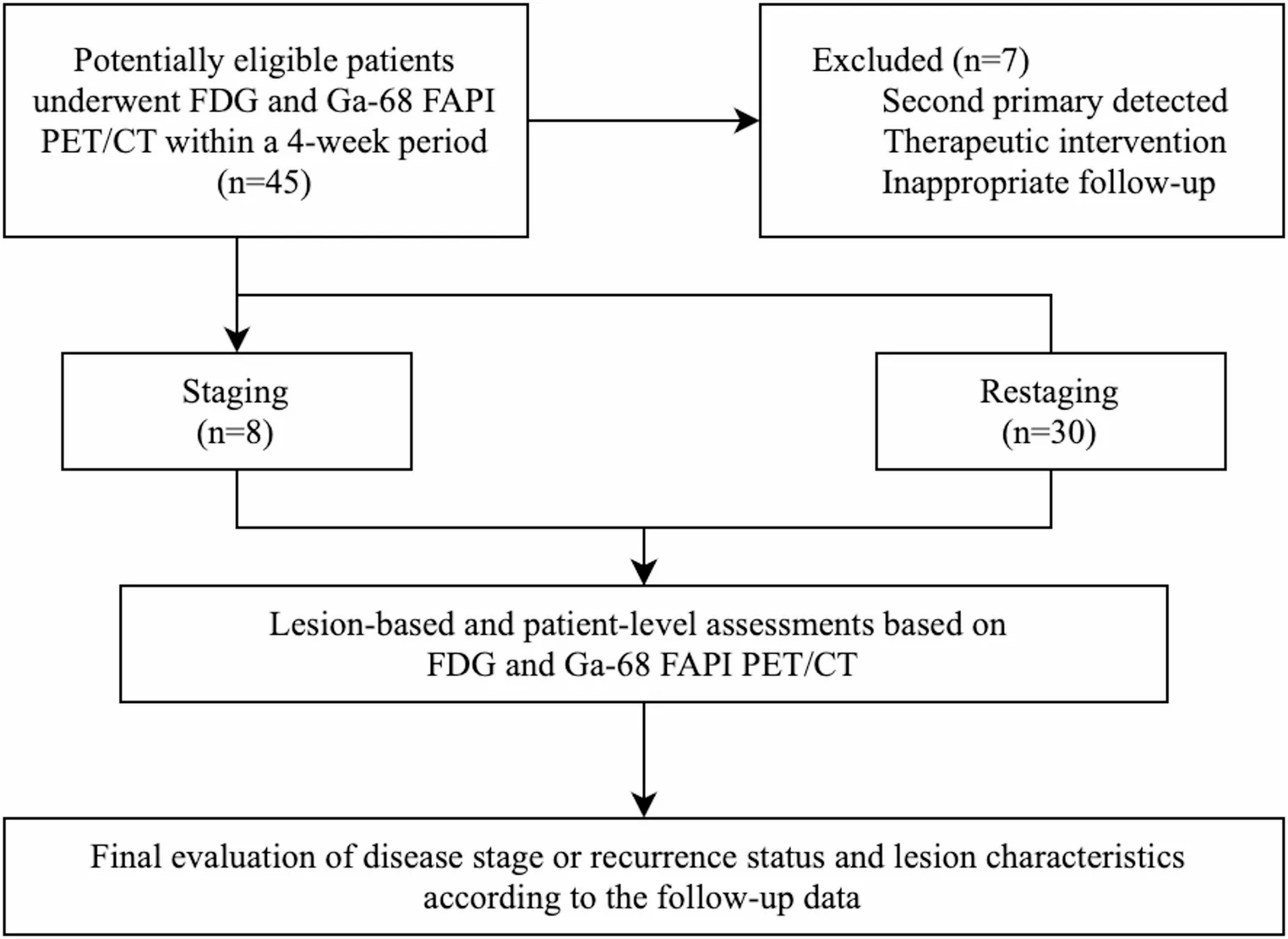
The scheme of the study design

Adult patients (≥ 18 years) with histologically confirmed gastrointestinal malignancies who were under care in the Oncology or Surgery Department were retrospectively enrolled. Eligible patients underwent both 18 F-FDG and 68Ga-FAPI PET/CT within a 4-week interval for staging or restaging based on clinical, imaging, and laboratory findings. Exclusion criteria included patients with a known second primary malignancy or those who had received any therapeutic intervention between the two PET/CT examinations, to avoid treatment-related alterations in tracer uptake and disease extent. Additionally, patients with insufficient follow-up data after PET imaging were excluded from this study to ensure precise reference-standard characterization of identified lesions in these examinations (Fig. 1).

Demographic, clinical, histopathological, and imaging data were retrieved from the institutional hospital information system. Both patient-level and lesion-based analyses were conducted to compare the diagnostic performance of FDG and FAPI PET/CT.

Only clinical and imaging information available at the time of PET/CT acquisition was accessible to the interpreters. After the image assessment was completed, follow-up results were obtained from the institutional database.

### PET/CT acquisition protocols

18 F-FDG PET/CT was performed after a minimum 6-hour fast, with plasma glucose < 150 mg/dL. Patients received a dose of 3.7 MBq/kg (200–370 MBq) and image acquisition was initiated approximately 60 ± 5 min after tracer injection.

68Ga-FAPI-04 PET/CT was performed without restriction on plasma glucose levels, provided that at least 4 h of fasting were ensured for optimal evaluation of the abdominal cavity. Images were acquired at two time points: early (10 min) and delayed (60 ± 5 min) after tracer injection (100–200 MBq). A dual-time-point FAPI imaging approach was taken to explore the tracer kinetics, given the absence of a standardized optimal imaging time for FAPI PET/CT in gastrointestinal malignancies.

All examinations covered the vertex-to-mid-thigh region and were reconstructed using an ordered-subsets expectation-maximization (OSEM) algorithm.

### Image interpretation

All PET/CT images were reviewed on a dedicated workstation (Advantage Workstation 4.7, GE Healthcare). PET, fused PET/CT, and maximum intensity projection images were evaluated in axial, coronal, and sagittal planes.

FDG PET/CT images were interpreted according to the European Association of Nuclear Medicine (EANM) guidelines for oncologic PET/CT reporting [20], and FAPI PET/CT images were evaluated following the Society of Nuclear Medicine and Molecular Imaging (SNMMI) Procedure Standard/EANM Practice Guideline recommendations [17].

For both tracers, focal uptake exceeding background activity not attributable to physiological biodistribution was included in the assessment. Additionally, lesions meeting the response evaluation criteria in solid tumors (RECIST) criteria for the anatomical component were recorded even in the absence of tracer uptake, consistent with routine clinical reporting practice.

Each FDG and FAPI PET/CT examination was interpreted independently for each patient, taking into account patient history, prior imaging findings, and known treatments up to the scan date; however, the reviewers did not have access to information from subsequent follow-up studies.

### Lesion-based analysis

For each detected lesion, the following data were recorded: anatomical location, maximum diameter, morphological correlate, and lesion characterization (primary tumor, metastasis, or nonspecific). Maximum standardized uptake values (SUVmax) were recorded separately for early and delayed FAPI PET/CT images.

Lesions were classified independently by two experienced nuclear medicine specialists using a four-point Likert scale: 0 = not detected, 1 = benign, 2 = indeterminate, and 3 = malignant.

Lesion tables for FDG and FAPI PET/CT were generated separately. A composite reference standard was established for each lesion using all available follow-up information. Histopathological confirmation was considered the preferred reference whenever available. However, as histological verification of all detected lesions was not feasible in routine clinical practice, lesion characterization was established based on a combination of serial imaging findings, surgical reports, laboratory results, clinical course, and multidisciplinary consensus. Lesions demonstrating unequivocal progression on follow-up imaging, treatment-related regression, or other imaging and clinical findings consistent with malignancy were considered malignant. Lesions that remained stable during follow-up without evidence of malignant behavior, resolved spontaneously, or showed findings consistent with benign etiologies were classified as benign. For each lesion, the method used for the final decision was also recorded. Subsequently, all three lesion tables, including FDG, FAPI, and follow-up results, were merged.

Semiquantitative analysis was performed for lesions with visually discernible tracer uptake on the corresponding PET/CT acquisition. Non-detected or tracer-negative lesions were excluded from SUVmax-based comparative interpretation to avoid misleading quantitative estimates. Therefore, FDG and FAPI were primarily compared for lesion detection and characterization, whereas SUVmax-based analyses focused on measurable tracer-positive lesions and early-to-delayed FAPI uptake dynamics.

Background activity was measured from the same reference region on both early and delayed FAPI images using a fixed spherical volume-of-interest placed in the ascending aorta. The tumor-to-background ratio (TBRmax) was defined as the ratio of lesion SUVmax to blood-pool SUVmean.

### Patient-level analysis

In addition to lesion-based analyses, complementary patient-level assessments were also performed. The disease stage was determined for each modality based on the current TNM Classification for Malignant Tumors. For restaging examinations, the presence of recurrent or metastatic disease was assessed. Additionally, PCI scores and the total number of detected malignant lesions for each patient were calculated separately from FDG and FAPI imaging findings.

The diagnostic value of FAPI PET/CT compared with FDG PET/CT, and its potential impact on patient management, were evaluated by a multidisciplinary team comprising an oncologist, surgeon, and nuclear medicine specialist, subsequently validated against the reference standard.

### Statistical analysis

Statistical analyses were performed using IBM SPSS Statistics version 29 (IBM Corp., Armonk, NY, USA) under the guidance of an expert from the Biostatistics Department. Continuous variables were presented as mean ± standard deviation or median, depending on the distribution, whereas categorical variables were reported as counts and percentages. Normality was assessed using the Shapiro-Wilk test.

Comparisons between continuous variables were performed using paired-samples t-tests or Wilcoxon signed-rank tests. Repeated measurements across PET/CT acquisitions were compared using the Friedman test. Comparisons of PET variables based on lesion size were performed using the Mann-Whitney U test. Differences in malignancy detection among imaging modalities and reference tests were assessed using Cochran’s Q test with post hoc comparisons. The association between change in SUVmax and lesion malignancy was analyzed using binary logistic regression.

The diagnostic performance of the examinations for lesion detection and recurrence assessment was evaluated using detection rate, accuracy, Cohen’s kappa coefficients, and receiver operating characteristic (ROC) curve analysis. A p-value < 0.05 was considered statistically significant, with Bonferroni correction applied for multiple comparisons.

## Results

### Patient characteristics

A total of thirty-eight patients with histologically confirmed gastrointestinal malignancies who underwent both 18 F-FDG and 68Ga-FAPI PET/CT for initial staging or restaging were included in the analysis. Seven patients were excluded from the study: 1 had a second primary malignancy, 4 had insufficient follow-up data, and 2 received treatment between investigations. The study population comprised 26 males and 12 females, with a median age of 59 years (range, 36–79). The cohort consisted of 8 patients undergoing staging (21%) and 30 for restaging (79%). The median interval between FDG and FAPI PET/CT examinations was 8.5 days (range, 1–28 days), and no therapy was administered between scans. Primary tumor origins included mostly gastric (37%) and colorectal (31%), while the esophagus, pancreas, liver, gallbladder, peritoneum, and appendix were also present. Histopathologically, adenocarcinomas accounted for the vast majority of patients in the study; mucinous and signet-ring cell carcinomas, known for low FDG avidity, were present in 32% of the cohort. Patient demographics and baseline clinical characteristics are summarized in Table 1.

**Table 1:** Patient demographics and clinical characteristics

Variable	
**Number of patients**, n	38
**Age (years)**, mean ± SD	59.2 ± 12.3
**Sex**, n (%)	
Male	26 (68%)
Female	12 (32%)
**Imaging indication**, n (%)	
Staging	8 (21%)
Restaging	30 (79%)
**Primary tumor origin**, n (%)	
Stomach	14 (37%)
Colon	12 (31%)
Pancreas	4 (10%)
Liver	3 (8%)
Appendix	2 (5%)
Esophagus	1 (3%)
Gallbladder	1 (3%)
Peritoneal	1 (3%)
**Histopathological subtype**, n (%)	
Adenocarcinoma	22 (58%)
Mucinous adenocarcinoma	6 (16%)
Signet-ring cell	6 (16%)
Other	4 (10%)
**Time interval between FDG and FAPI PET/CT**, days, median (range)	8.5 (1–28)

SD: Standard deviation, PET/CT: Positron emission tomography/computed tomography

Data are presented as median (range) or number (percentage), as appropriate

### Patient-level analysis

**Fig. 2:**
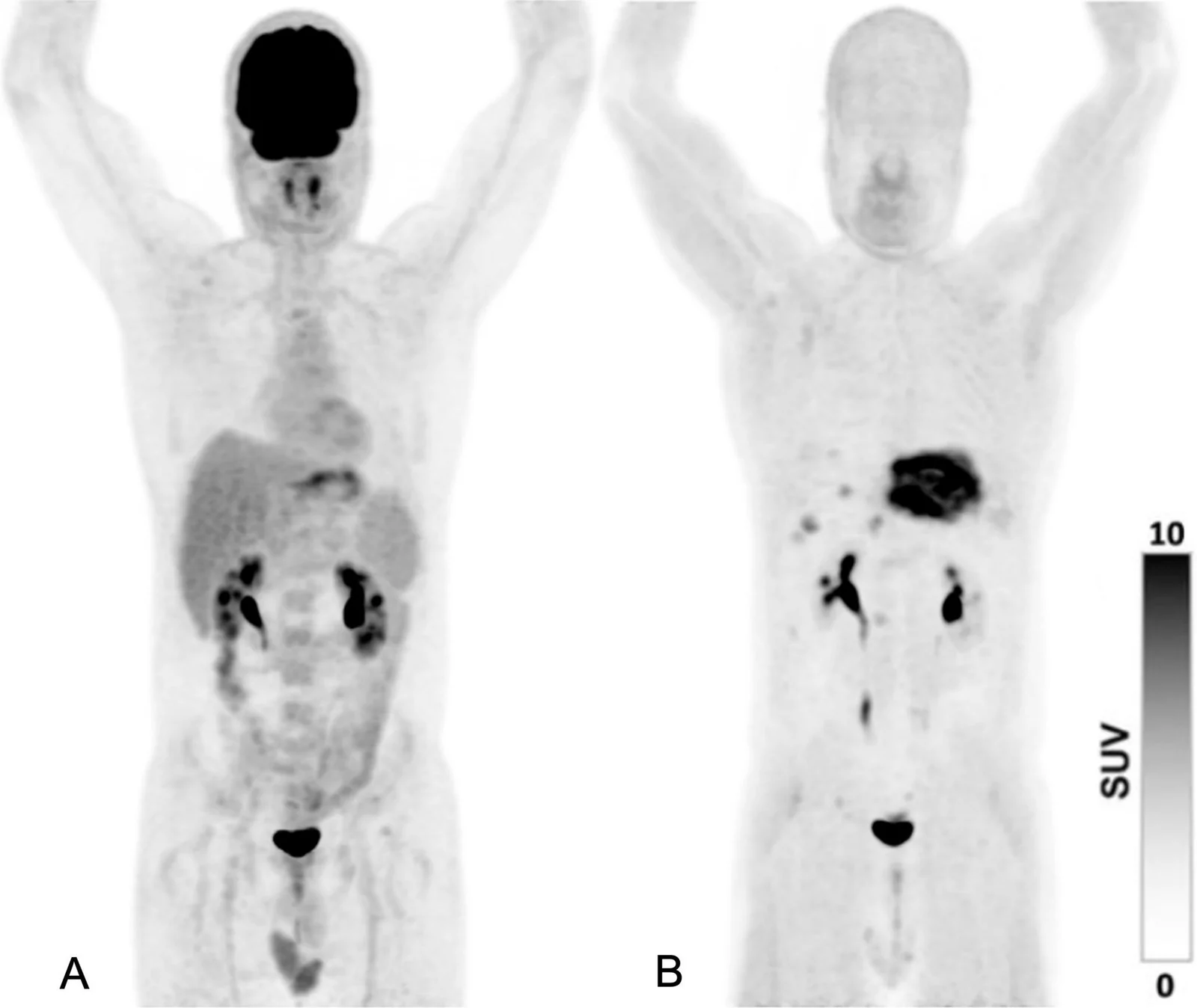
Representative maximum intensity projection images of FDG (**a**) and FAPI PET/CT (**b**) in a patient with gastric cancer. FDG PET/CT suggested localized disease, whereas FAPI PET/CT revealed extensive peritoneal implants consistent with peritoneal carcinomatosis, leading to stage migration and change of patient management

Among the eight patients undergoing initial staging, FAPI PET/CT upstaged the disease in five patients (63%) compared to FDG PET/CT. All FAPI-derived staging results aligned with follow-up outcomes. Follow-up data indicated recurrent disease in 20 of 30 patients within the restaging cohort. FAPI PET/CT accurately determined the recurrence status in 28 out of 30 patients (93%), whereas FDG PET/CT achieved success in 18 of 30 cases (60%). Based on predefined criteria, FAPI PET/CT provided additional diagnostic information over FDG PET/CT in most patients. Malignant lesions were detected exclusively by FAPI PET/CT in 10 patients despite negative FDG PET/CT findings; in 13 other patients, FAPI PET/CT showed either more widespread disease or additional malignant sites than FDG PET/CT. In 11 cases, FAPI PET/CT helped to clarify suspicious findings in other examinations and rule out recurrent disease. Only 4 patients (10%) received no additional diagnostic benefit from FAPI PET/CT. Figure 2 provides an example of a comparison between modalities.

Changes in patient management attributable to FAPI PET/CT findings were observed in 63% of patients, including initiation of treatment, adjustment of ongoing therapy, or avoidance of unnecessary invasive procedures. Information on the staging, recurrence status, diagnostic contributions, and their impact on disease management is provided in Table 2 for staging patients and Table 3 for restaging patients. The reference standard consisted of histopathological verification, when available (20/38 patients), and a composite assessment based on serial imaging, clinical follow-up, treatment response, and multidisciplinary review for the remaining patients. Agreement analysis demonstrated concordance between FAPI PET/CT findings and the reference standard (κ = 0.916, *p* < 0.001), while FDG PET/CT showed lower agreement (κ = 0.320, *p* = 0.002).

**Table 2:** Staging outcomes and implications according to patients

Stage Assessment							
Case #	Primary Disease	Subtype	Stage (TNM)			Diagnostic Contribution	Effect on Management
			FDG	FAPI	Final		
2	Esophagus	SCC	III	III	III	No	No
3	Pancreas	Adenoca	III	IV	IV	Yes	Yes
4	Gastric	Adenoca	I	IV	IV	Yes	Yes
5	Liver	HCC	X	II	II	Yes	No
11	Appendix	Mucinous	X	X	X	Yes	No
17	Appendix	Mucinous	X	IV	IV	Yes	Yes
29	Gastric	Signet-cell	I	II	II	Yes	No
31	Colon	Mucinous	II	II	II	Yes	No

*X means the test showed no disease

*Staging results were pooled for analysis. Diagnostic contribution was defined as improved detection or characterization of malignant disease on FAPI PET/CT compared with FDG PET/CT. The impact on patient management was assessed retrospectively by a multidisciplinary team, examining changes in clinical decision-making

**Table 3:** Restaging results by imaging findings

Restaging Assessment						
Final Diagnosis	FDG		FAPI		Diagnostic Contribution	Effect on Management
	-	+	-	+		
Recurrence Absent (*n* = 10)	7/10	3/10	9/10	1/10	9/10	7/10
Recurrence Present (*n* = 20)	9/20	11/20	1/20	19/20	18/20	14/20

*Restaging results were pooled for analysis. Diagnostic contribution was defined as improved detection or characterization of malignant disease on FAPI PET/CT compared with FDG PET/CT. The impact on patient management was assessed retrospectively by a multidisciplinary team, examining changes in clinical decision-making

### Lesion-based analysis

A total of 195 lesions were identified across all patients in PET/CT assessments, of which 135 were confirmed as malignant based on follow-up data. FDG PET/CT detected 46 malignant lesions (34%), while FAPI PET/CT correctly identified 116 malignant lesions (86%). In the FAPI evaluation, 11 of the remaining 19 malignant lesions were not detected, 7 could not be characterized, and 1 was misclassified as benign. Conversely, out of the 135 malignant lesions, 64 (47%) were not detected on FDG; one lesion was assessed as benign; and for 24 lesions (18%), the benign/malignant distinction could not be determined. Lesion characteristics by modality and results are shown in Table 4. Lesion-based agreement with the reference standard was significantly higher for FAPI PET/CT (κ = 0.644, *p* < 0.001) than for FDG PET/CT (κ = 0.206, *p* < 0.001). ROC analysis demonstrated superior diagnostic performance of FAPI PET/CT (AUC = 0.894) compared with FDG PET/CT (AUC = 0.682). Malignant lesion detection rate and overall diagnostic accuracy were 85.9% and 83.1% for FAPI PET/CT, and 34.0% and 48.7% for FDG PET/CT, respectively.

**Table 4:** Lesion characteristics by modalities and results

Imaging Modality	Likert Score	Final Diagnosis		Total
		Benign	Malignant	
**FDG PET/CT**	0 – Not Detected	26	64	90
	1 – Benign	23	1	24
	2- Indeterminate	8	24	32
	3- Malignant	3	46	49
	**Total**	**60**	**135**	**195**
**FAPI PET/CT**	0 – Not Detected	8	11	19
	1 – Benign	38	1	39
	2- Indeterminate	8	7	15
	3- Malignant	6	116	122
	**Total**	**60**	**135**	**195**

*The final diagnosis was determined based on histopathology when available or on clinical and imaging follow-up. Lesions not visualized on a given modality were classified as “not detected” for that modality. Indeterminate lesions were those that could not be definitively characterized as benign or malignant at the time of imaging

### Lesion based evaluation and peritoneal disease assessment

FAPI PET/CT outperformed FDG PET/CT in detecting primary tumors, local recurrences, lymph node metastases, visceral metastases, and peritoneal implants. The most pronounced difference was observed for peritoneal implants, where FAPI PET/CT detected 71 of 77 lesions (92%), compared with 18 lesions (23%) detected by FDG PET/CT.

The median number of malignant lesions per patient was 1 (range, 0–10) for FDG PET/CT and 1.5 (range, 0–15) for FAPI PET/CT. Median peritoneal carcinomatosis index (PCI) scores were 0 (range, 0–16) for FDG PET/CT and 1 (range, 0–25) for FAPI PET/CT. Regarding the number of malignant lesions detected and PCI scores for each patient, FAPI yielded higher values than FDG, with a statistically significant difference between the two tests (*p* < 0.001). See Table 5 for a comparison of FDG-FAPI for detected malignant lesions and PCI score.

**Table 5:** Comparison of FDG-FAPI for detected malignant lesions and PCI score

Variable	FDG< FAPI (*n*)	FDG> FAPI (*n*)	FAPI = FDG (*n*)	Z Score**	*p* Value
**Detected Malignant Lesions**	20	3	15	-3.449	< 0.001
**PCI Score**	17	1	20	-3.574	< 0.001

*Abbreviations: n, number of patients; FDG, 18 F-Fluoro-deoxyglucose PET/CT; FAPI, 68Ga-Fibroblast Activating Protein Inhibitor-04 PET/CT

**Z-scores based on the Wilcoxon signed-rank test comparing lesion counts on a patient basis

### Semiquantitative analysis

Semiquantitative analysis demonstrated significant differences in tracer uptake across imaging modalities and acquisition times for FAPI images. Beyond its superior lesion detection rate, FAPI PET/CT also showed significantly higher tracer uptake than FDG PET/CT among forty-three malignant lesions only visualized on both examinations, with median SUVmax were 5.58 for early FAPI PET/CT, 8.35 for delayed FAPI PET/CT, and 4.55 for FDG PET/CT (*p* < 0.001).

**Fig. 3:**
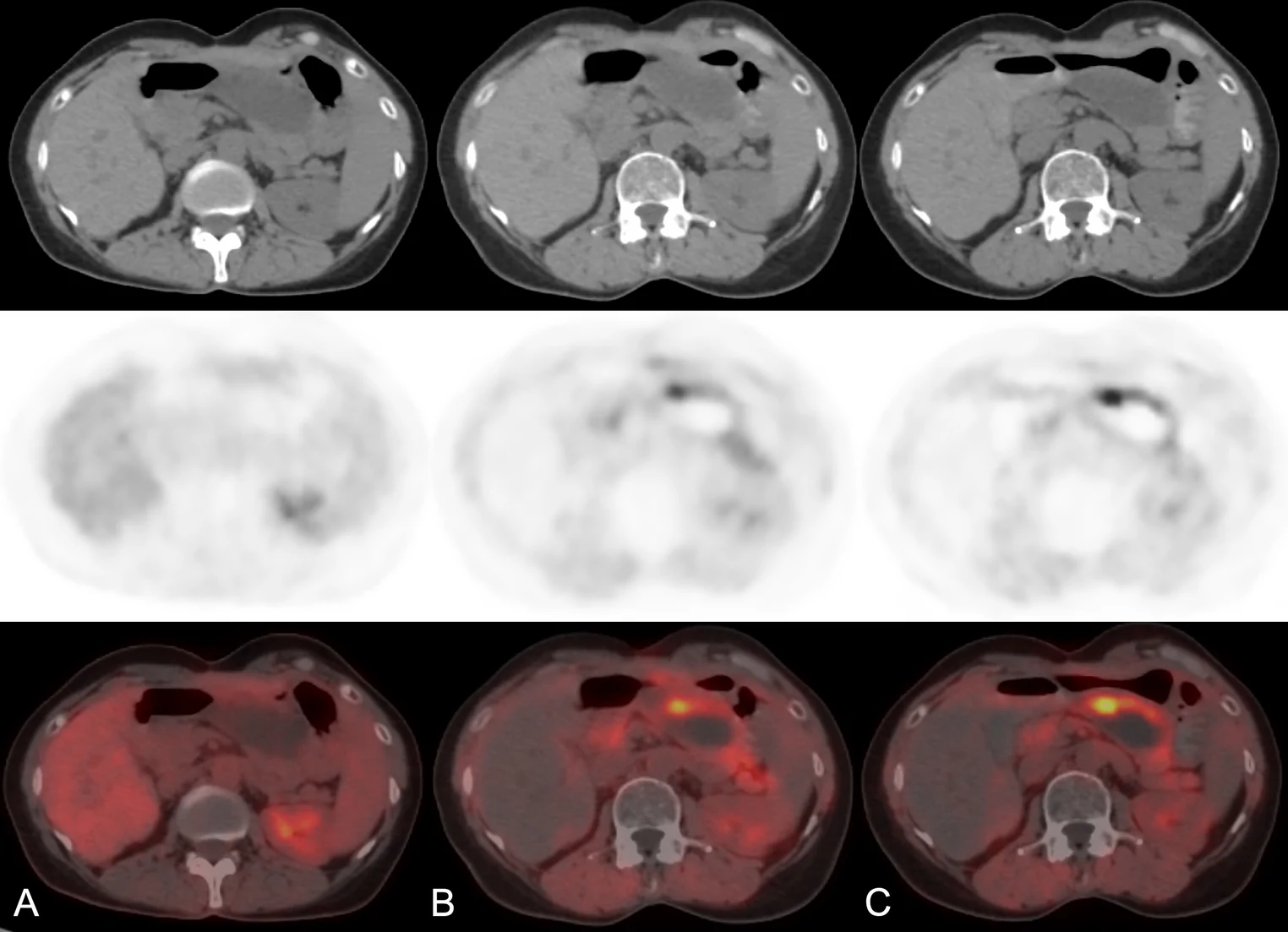
A patient diagnosed with gastric cancer, who was evaluated for recurrent disease due to the appearance of a solitary cystic lesion in the lesser omentum that grew over time. FDG PET/CT (**a**) and FAPI PET/CT (**b** for 10th minute and **c** for 60th minute) images are shown. No significant FDG uptake was detected, and the uptake intensity in the lesion gradually increased between the 10th and 60th minute images. Surgical excision and HIPEC were performed; histopathology confirmed mucinous adenocarcinoma infiltration

Median SUVmax for malignant lesions was progressively higher on early (median SUVmax was 4.27 for 10th min) and delayed (median SUVmax was 5.28 for 60th min) FAPI PET/CT imaging (*p* < 0.001). In addition, the TBRmax was calculated to provide a background-corrected measure of tracer uptake. Similar to the SUVmax findings, TBRmax values were also significantly higher on delayed FAPI PET/CT compared with early imaging (median TBRmax: 4.59 vs. 2.72, *p* < 0.001). Lesion-based comparisons revealed a consistent increase in SUVmax and TBR from early to delayed FAPI acquisitions across the majority of malignant lesions (Fig. 3). Post hoc analysis revealed statistically significant differences across all pairwise comparisons after Bonferroni correction.

**Fig. 4:**
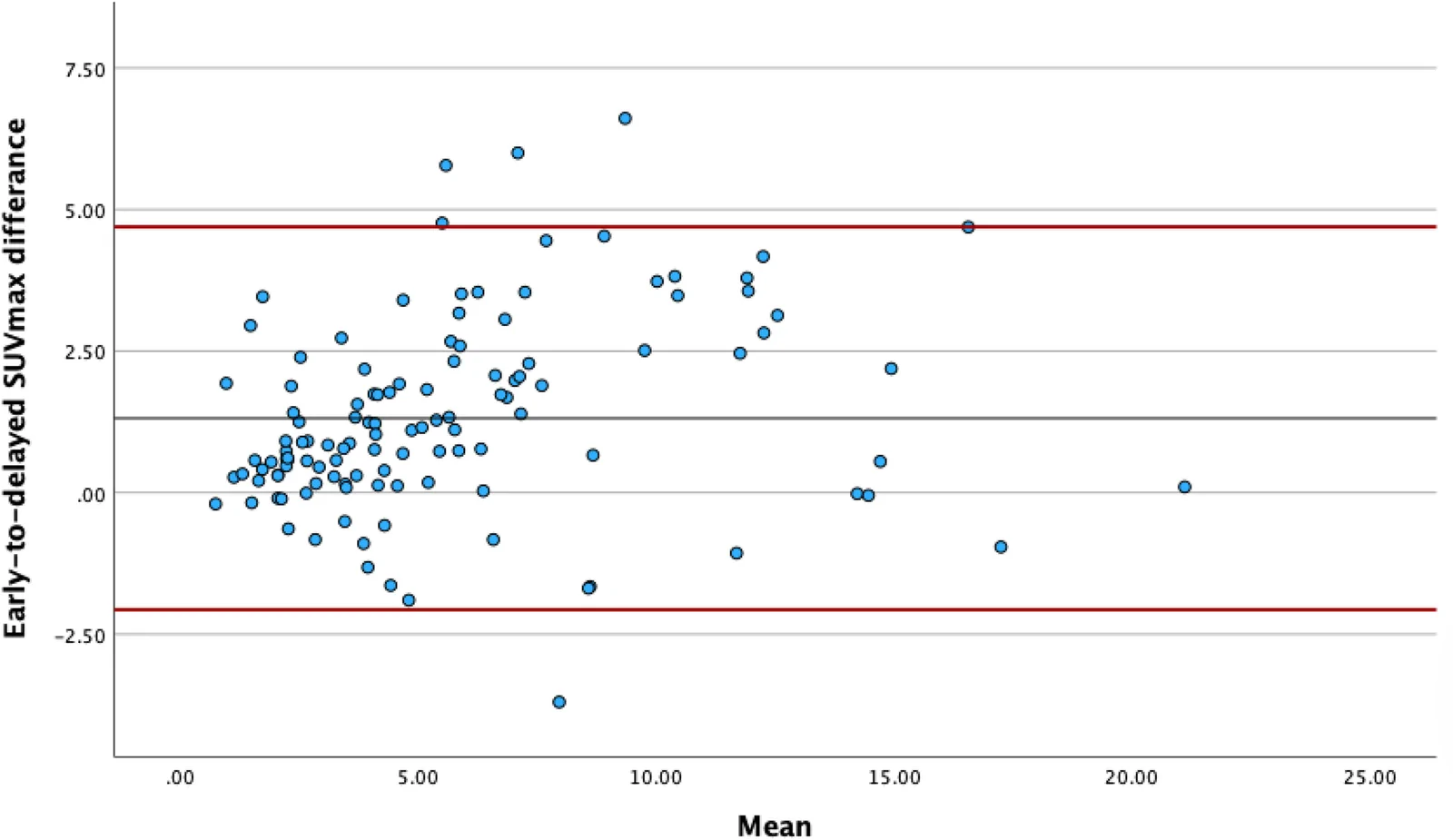
Bland-Altman Analysis: Difference between early and late FAPI SUVmax

Bland–Altman analysis showed a systematic positive bias in delayed FAPI SUVmax relative to early FAPI measurements, indicating greater tracer accumulation at 60 min in malignant lesions (Fig. 4). The mean SUVmax difference was 1.31 between delayed and early FAPI images (CI: -2.06; +4.69). The magnitude of this increase varied among lesions but was reproducible within patients, supporting the added value of delayed FAPI imaging for semiquantitative assessment. Binary logistic regression analysis revealed that increasing delayed-to-early FAPI SUVmax differences were independently associated with a higher probability of malignancy (odds ratio = 1.014, *p* = 0.008). Delayed FAPI PET/CT yielded higher SUVmax values than early FAPI PET/CT across most lesion categories, particularly in primary lesions and peritoneal implants (Table 6). Conversely, in benign lesions, no significant difference was observed between early and delayed FAPI PET/CT after correction for multiple comparisons. Therefore, physiological pancreatic uptake, which represents background activity, showed significantly higher SUVmax values at 10 min (median 2.39) than at 60 min (median 1.91) post-injection (*p* < 0.001).

**Table 6:** Malignant lesion detection rates and characteristics

Lesion Type	Detection Rates		Diameter (mm)	SUVmax	
	FDG	FAPI		FAPI (10-min)	FAPI (60-min)
**Primary**	6/9	8/9	45 (27–127)	6.64 (5.08–21.05)	11.17 (3.85–21.15)
**Local Recurrence**	2/4	4/4	38.5 (16–50)	4.93 (4.5-14.21)	6.48 (4-18.9)
**Lymph Node**	5/13	11/13	10.5 (8–27)	4.27 (1.9-14.43)	4.48 (2-14.98)
**Visceral**					
***Liver***	*7/14*	*9/14*	*11 (7–25)*	*4.82 (1.64–12.22)*	*5.25 (2.18–11.15)*
***Lung***	*5/7*	*5/7*	*6*.*5 (5–12)*	*2.09 (1.03–14.23)*	*1.9 (1.02–14.21)*
***Other***	*3/11*	*8/11*	*30 (12–41)*	*4.45 (1.99–9.81)*	*6.07 (2.46–8.54)*
**Implant**	18/77	71/77	14 (5–51)	3.49 (1.07–14.48)	4.95 (1.26–14.43)
**Overall**	46/135	116/135	14 (5-127)	4.27 (1.03–21.05)	5.28 (1.02–21.15)

Lesion size was measured as the maximum axial diameter on the CT component of PET/CT. SUVmax values were obtained from standardized volume-of-interest analyses on FDG PET/CT and early (10th min) and delayed (60th min) FAPI PET/CT for tracer-positive lesions. Continuous variables are presented as median (range). For peritoneal lesions, measurements were performed on the most representative lesion per anatomical region

### Lesion size, histopathology, and SUVmax dynamics

Lesion-based analysis revealed a significant association between lesion size and tracer detectability. A notable retention of tracer was observed at malignant lesions on 60-minute FAPI imaging, which were closely related to lesion size. When lesions were stratified by size, SUVmax on delayed FAPI PET/CT remained significantly higher than those on early PET/CT in high-volume lesions. Specifically, lesions greater than 10 mm showed a statistically significant rise in SUVmax on delayed imaging compared to smaller lesions (*p* = 0.001).

Subgroup analysis by histopathological subtype indicates consistent tracer behavior across various gastrointestinal malignancies, despite heterogeneity observed in both absolute SUVmax values and early-to-delayed FAPI uptake changes. The Kruskal-Wallis test was used to evaluate changes in SUVmax from early to delayed measurements across histopathological subtypes in malignant lesions. Although SUVmax increased in the 60-minute images across all subgroups, no statistically significant differences were observed among them (*p* = 0.310).

The differences in malignant lesion detection across pathological subtypes were also assessed. Statistically significant differences were identified between FDG, FAPI, and outcome findings (*p* < 0.001). FDG and outcome findings differed significantly across all subtypes, whereas no significant difference was found between FAPI and outcome findings. Notably, higher P-values, indicating superior diagnostic performance, were observed in comparisons of outcome and FAPI findings for mucinous and signet-ring cell carcinoma versus the other group, comprising esophageal SCC, HCC, and cholangiocellular carcinoma.

## Discussion

### FDG versus FAPI PET/CT: overall diagnostic performance

PET/CT plays a central role in oncologic imaging, and FDG remains the most widely used PET radiopharmaceutical in clinical practice. Previous reviews have highlighted multiple limitations of FDG PET/CT in various malignancies and have compared its diagnostic performance with 68Ga-FAPI PET/CT [16, 21]. However, in this study, we simultaneously evaluated malignant and indeterminate lesions while also addressing the time-dependent behavior of uptake patterns. In this context, our study provides a comprehensive comparison of FDG and FAPI PET/CT at both the patient and lesion levels. In our cohort, most patients underwent restaging, reflecting institutional practice in which FAPI PET/CT is predominantly used as a problem-solving tool in patients with suspected biochemical recurrence or inconclusive imaging findings. This clinical context explains the relatively small proportion of patients imaged for primary staging and the low recurrence rates at follow-up.

FAPI PET/CT showed better agreement with final clinical outcomes (κ = 0.91) than FDG PET/CT (κ = 0.32) for stage or recurrence status. Only two patients were incorrectly classified by FAPI PET/CT. One involved a patient with colorectal cancer and a history of local ablative treatment for liver metastases, where both FDG and FAPI showed false-positive uptake corresponding to granulomatous inflammation on histopathology. These findings highlight the importance of careful interpretation of FAPI PET/CT, especially in previously treated areas, to maintain diagnostic accuracy and confidence. FAPI PET/CT also provided additional diagnostic information beyond FDG PET/CT in 90% of patients. Importantly, FAPI findings led to changes in clinical management in 63% of patients. In 37% of cases, treatment was initiated or modified based on lesions detected exclusively on FAPI PET/CT. Results are consistent with the literature for gastrointestinal cancer patients imaged with FAPI compared with FDG [12].

### Peritoneal disease assessment and PCI analysis

Accurate assessment of disease burden is particularly relevant in peritoneal carcinomatosis, where the peritoneal cancer index (PCI) has prognostic value. Although PCI is traditionally determined intraoperatively, preoperative estimation using imaging modalities is increasingly applied [18, 19]. Prior studies have demonstrated that FAPI-based PCI assessment is more accurate than FDG-based evaluation across various malignancies [14, 22–23]. In this study, to make the comparison more robust, PCI scores were evaluated in a cohort encompassing a broad spectrum of gastrointestinal cancers.

In our cohort, median PCI scores were significantly higher when calculated from FAPI PET/CT than from FDG PET/CT (1 vs. 0, *p* < 0.001). The number of detected malignant lesions per patient was also significantly higher with FAPI PET/CT (median: 1.5 vs. 1; *p* < 0.001). Low median values likely reflect the low pretest probability for peritoneal carcinomatosis of the cohort. Follow-up data also support this proposition: 10 of 30 patients who underwent restaging didn’t relapse. Nonetheless, our results support the superior detection rate of FAPI PET/CT for peritoneal involvement and malignant lesions.Lesion-based analysis and dual-time-point FAPI imaging.

Lesion characterization was performed in a clinically realistic, context-based manner, accounting for uptake patterns and available clinical information. The four-point scale has been designed to align with clinical practice and to minimize false-positive and false-negative results. Lesions with uptake patterns potentially attributable to inflammatory or fibrotic processes were carefully categorized as indeterminate.

Lesion-based analysis further highlighted the advantages of FAPI PET/CT. Agreement with the final diagnosis was better for FAPI PET/CT (κ = 0.64) than for FDG PET/CT (κ = 0.20). The detection rate for malignant lesions was markedly higher with FAPI (86%) than with FDG (34%), consistent with previous reports [12, 14]. Sensitivity and specificity were not calculated due to incomplete histopathological verification.

A notable advantage of FAPI PET/CT was its improved lesion characterization. The proportion of indeterminate lesions was lower with FAPI than with FDG, and indeterminate lesions on FAPI were less likely to be malignant than those classified as indeterminate on FDG.

**Fig. 5:**
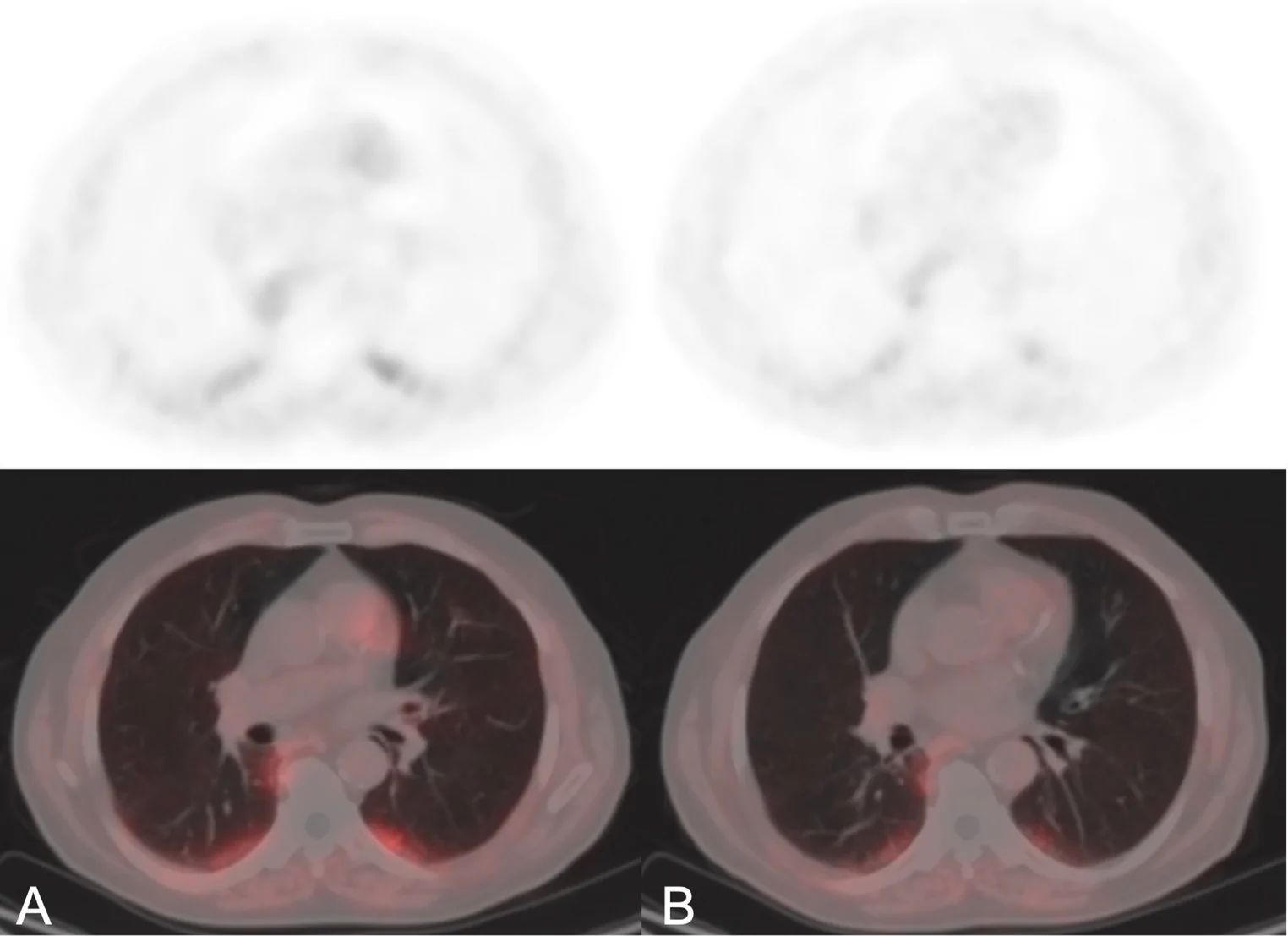
An example of benign 68Ga-FAPI uptake in the lungs shows that intensity decreased over time between the 10th-minute (**a**) and 60th-minute (**b**) images

Semi-quantitative analysis demonstrated that malignant lesions showed a consistent and significant increase in SUVmax between early (10 min) and delayed (60 min) FAPI acquisitions, whereas benign lesions exhibited stable uptake over time (Fig. 5). Moreover, logistic regression analysis demonstrated that increasing early-to-delayed SUVmax differences were associated with a higher probability of malignancy. This divergent temporal behavior suggests that dual-time-point FAPI imaging may improve discrimination between malignant and benign lesions beyond that achievable with static imaging alone.

Importantly, the increase observed on delayed imaging was confirmed not only by SUVmax measurements but also by TBRmax analysis. Delayed FAPI PET/CT yielded significantly higher TBRmax values for malignant lesions than early imaging (median TBRmax: 4.59 vs. 2.72, *p* < 0.001), indicating that the observed uptake enhancement remained significant after correction for background activity. These findings further support the superiority of delayed imaging for lesion characterization. In parallel, physiological pancreatic uptake, measured separately to characterize the temporal biodistribution of FAPI and to demonstrate changes in normal-organ activity, decreased significantly over time, thereby improving lesion-to-background contrast on delayed images. These findings align with prior preclinical and clinical observations indicating washout of FAPI from non-target tissues over time, concomitant with an increase in its uptake in tumoral tissues [13, 24]. Taken together, our results support the notion that delayed FAPI imaging enhances lesion conspicuity, providing a strong rationale for incorporating delayed acquisitions into clinical FAPI PET/CT protocols.

### Effect of lesion qualification on detection

FAPI PET/CT detected more malignant lesions across all lesion categories, including primary tumors, local recurrences, lymph nodes, visceral metastases, and peritoneal implants, although SUVmax differences did not reach statistical significance in some subgroups. Interestingly, FDG showed higher SUVmax values only in pulmonary metastases, contrary to prior studies [12]. FDG uptake intensities were higher in metastases than in FAPI (3.65 vs. 2.09, respectively). This difference suggests factors such as the small sample size, the prominence of radionuclide-related (68Ga and 18 F) spatial-resolution differences in small lesions (the smallest metastasis group, with a median of 6.5 mm), or the inability to standardize SUVmax measurements due to respiratory artifacts.

Another remarkable finding of this study is the demonstrated interaction between lesion size and FAPI tracer kinetics. Greater malignant lesions (> 10 mm) exhibited significantly higher early-to-delayed SUVmax increases than smaller lesions, a pattern not observed in benign lesions (*p* = 0.001). No significant differences were found for other lesion-size thresholds, such as 15 and 20 millimeters. It is well known that visibility is low, especially for very small lesions, due to the partial-volume effect [7]. However, when the same evaluation was performed for benign lesions, no statistically significant difference was found, so the partial-volume effect could not explain the finding. This observation suggests that lesion size may influence FAPI accumulation dynamics, potentially reflecting differences in stromal composition, tracer diffusion, or partial-volume effects in small-volume disease.

Histopathological subtype analysis revealed that FAPI PET/CT outperformed FDG PET/CT across all tumor types, with particularly pronounced differences in mucinous and signet-ring cell carcinomas. While FDG performed relatively better in certain non-adenocarcinoma subtypes, FAPI PET/CT showed greater overall consistency with reference standards. No significant association was observed between histopathological subtype and the magnitude of SUVmax change between early and delayed imaging.

### Limitations and future perspectives

This study presents notable strengths, including a comprehensive comparison of 18 F-FDG and 68Ga-FAPI PET/CT at both patient and lesion levels, minimizing interindividual variability. It uniquely incorporates dual-time-point FAPI imaging to evaluate early- and delayed-tracer uptake dynamics, thereby addressing time-dependent changes in SUVmax that have been underexplored in prior studies. Including both malignant and indeterminate lesions allows a realistic assessment of diagnostic performance in clinical practice. Additionally, lesion-based analyses were paired with clinically relevant outcome measures, such as staging accuracy and peritoneal cancer index assessment. However, limitations include a retrospective design that may introduce selection bias, a small sample size that limits generalizability, and variability in tumor origins that could skew tracer uptake. Some lesions lacked histopathological confirmation, relying instead on imaging and clinical follow-up. Despite these limitations, our findings add meaningful evidence to the growing body of literature supporting the clinical utility of FAPI PET/CT.

## Conclusion

68Ga-FAPI PET/CT outperformed FDG PET/CT for both staging and restaging of gastrointestinal malignancies, providing superior lesion detection, more accurate assessment of disease extent, and stronger concordance with follow-up data. Compared with FDG PET/CT, FAPI PET/CT identified more malignant lesions, yielded higher peritoneal cancer index scores, and resulted in fewer indeterminate findings, thereby reducing diagnostic uncertainty.

A major strength of this study is the evaluation of dual-time-point FAPI imaging. Delayed imaging was associated with a significant increase in SUVmax in malignant lesions, whereas benign lesions showed no comparable change, improving discrimination between malignant and non-malignant findings. Notably, this temporal uptake pattern was influenced by lesion size, with larger lesions demonstrating more pronounced SUVmax increases on delayed images. Furthermore, increasing early-to-delayed SUVmax differences were independently associated with a higher probability of malignancy.

Taken together, these findings indicate that dual-time-point 68Ga-FAPI PET/CT not only enhances lesion detectability but also improves lesion characterization and diagnostic confidence, supporting its role as a problem-solver and a complementary imaging strategy to FDG PET/CT in gastrointestinal malignancies.

## Data Availability

The datasets generated and/or analyzed during the current study are available from the corresponding author on reasonable request.
